# Mouse Tissues Express Multiple Splice Variants of Prominin-1

**DOI:** 10.1371/journal.pone.0012325

**Published:** 2010-08-20

**Authors:** Kristel Kemper, Marc J. P. M. Tol, Jan Paul Medema

**Affiliations:** LEXOR (Lab for Experimental Oncology and Radiobiology), Center for Experimental Molecular Medicine, Academic Medical Center, Amsterdam, The Netherlands; Lehigh University, United States of America

## Abstract

Prominin-1, a heavily glycosylated pentaspan membrane protein, is mainly known for its function as a marker for (cancer) stem cells, although it can also be detected on differentiated cells. Mouse prominin-1 expression is heavily regulated by splicing in eight different variants. The function or the expression pattern of prominin-1 and its splice variants (SVs) is thus far unknown. In this study, we analyzed the expression of the prominin-1 splice variants on mRNA level in several mouse tissues and found a broad tissue expression of the majority of SVs, but a specific set of SVs had a much more restricted expression profile. For instance, the testis expressed only SV3 and SV7. Moreover, SV8 was solely detected in the eye. Intriguingly, prominin-1 knockout mice do not suffer from gross abnormalities, but do show signs of blindness, which suggest that SV8 has a specific function in this tissue. In addition, databases searches for putative promoter regions in the mouse prominin-1 gene revealed three potential promoter regions that could be linked to specific SVs. Interestingly, for both SV7 and SV8, a specific potential promoter region could be identified. To conclude, the majority of mouse prominin-1 splice variants are widely expressed in mouse tissues. However, specific expression of a few variants, likely driven by specific promoters, suggests distinct regulation and a potential important function for these variants in certain tissues.

## Introduction

The pentaspan membrane glycoprotein prominin-1 is widely studied as a stem cell surface marker, both in human [1], [2], [3], [4], [5], [6], [7], [8] and mouse [9], [10]. The human orthologue of prominin-1, called CD133 has been used as a marker in a variety of cancers to isolate cancer stem cells (CSCs) [1], [2], [3], [4], [5], [6] as well as hematopoietic stem cells [7], [8]. Even though CD133 is broadly used as a marker for (cancer) stem cells, the protein is also detected in more differentiated cell types [11], [12], [13], [14] and its exact function of CD133 on (cancer) stem cells remains enigmatic. However, it is quite evident that the expression of CD133 is heavily regulated. Human CD133 is reported to have seven splice variants (SVs) [15], which are under the control of five different promoters [16]. In addition, the CD133 protein is glycosylated, which, as we have shown before, is dependent on differentiation status of the (cancer) cell [15].

As in human, the murine prominin-1 is also expressed in differentiated cell types, shown by immunostainings for prominin-1 on mouse tissues [9], [14], [17] as well as by a prominin-1^LacZ/+^ mice, that express LacZ in all prominin-1 expressing cells [9], [10], [14]. In addition, colon tumors displayed an overall expression of prominin-1 [14], suggesting that also in mouse, prominin-1 expression is not restricted to a (cancer) stem cell state. Interestingly, prominin-1 did only mark the stem cell fraction in the small intestine [9], [10], indicating that the regulation of this protein might be different in this tissue.

Like the human orthologue, the prominin-1 protein can undergo heavy modification by glycosylation of its eight different N-linked glycosylation sites. In addition, the existence of a minimum of the eight SVs [10], [17] point to the possibility that mouse prominin-1 is highly regulated, although its promoters have not been identified yet.

The alternative splicing mostly affects the cytoplasmic C-terminus, resulting in four different C-terminal tails [17]. Differentially splicing of C-terminal tails suggest that prominin-1 SVs might interact with distinct cytoplasmic binding partners, potentially inducing specific signaling pathways and thereby exerting separate functions. Although cytoplasmic binding partners have not been identified for prominin-1, the C-terminal tail of SV3-5 resembles a class II PDZ-binding domain, while the C-terminal tail of SV1-2 and SV7-8 harbors characteristics of a class I PDZ-binding domain [18]. PDZ-binding domains are are thought to organize and regulate signaling complexes via protein-protein interactions. In agreement, a yeast two-hybrid screen showed that SV2 binds to a PDZ-domain containing novel splice variant of the glutamate receptor-interacting protein [18]. In human, Src and Fyn can phosphorylate two tyrosine residues on the C-terminal part of prominin-1 [19]. Altogether, this suggests that regulation of SV expression might influence the (signaling) function of the prominin-1 protein.

To gain more insight in the regulation of prominin-1 in mice, we decided to study the prominin-1 SVs by analyzing their expression pattern on protein and mRNA in several mouse tissues. We found that most SVs were expressed in all tissues. However, SV8 was specifically expressed in the eyes, whereas SV3 was only found in the eyes, testis and colorectal (CRC) cell line CMT93. In addition, SV7 was highly expressed in the testis. Interestingly, via database searches, we were able to identify a specific potential promoter region for both SV7 and SV8, suggesting that these two SVs have a more directed regulation and could therefore have a specific function.

## Materials and Methods

### Ethics Statement

Mice were maintained and experimented on in accordance with the guidelines of and after approval by the Dier Experimenten Commissie (DEC) of the Academic Medical Institute under permit number DIX100578.

### Mice tissues

Mouse tissues were obtained from C57BL/6J (WT) mice. The APC Min colon and polyp were obtained from C57BL/6J-ApcMin/J mice. After the animals were sacrificed, the tissues were retrieved and snap-frozen in liquid nitrogen.

### Cell lines

Mouse colorectal cell lines CMT93, C26, CC36 and MC38 [20] were cultured in Dulbecco's Modified Eagle Medium (DMEM, Invitrogen) containing 8% fetal calf serum (FCS), 2 mM glutamine, 100 U/ml penicillin, 100 µg/ml streptomycin, and 50 µM β-mercaptoethanol.

### PCR

Total RNA was isolated from homogenized tissues by TRizol extraction (Invitrogen) according to manufacturers' protocol. Quality and quantity of the RNA was measured on the Nanodrop ND-1000 Spectrophotometer (Nanodrop Technologies). With equal input of total RNA, cDNA was prepared with reverse transcriptase III (Invitrogen) using random primers. The PCR primers used for the splice variants are listed in Table 1. For GAPDH, the following promers were used: forward 5′- ATGTGTCCGTCGTGGATCTGA-3′and reverse 5′- ATGCCTGCTTCACCACCTTCT-3′. The PCRs were run at either 53°C or 54°C according to the following PCR program: 95°C for 4 min, then cycling for 26–34 cycles with 95°C for 30 s, 53/54°C for 30 s, 54°C for 30 s 72°C for 30 s followed by a final elongation step at 72°C for 10 min. PCRs were run on a 2% agarose gel.

**Table 1:** PCR primers used for discriminating prominin-1 splice variants.

Amplified region	SV	Forward primer	Reverse primer
exon 2 – exon 25	1/2	5′-CTGCATTCCATAACACTCCT-3′	5′-CAGGATTGTGAACACCATAT-3′
exon 3a – exon 4b	1/2	5′-GACATCTCAGTTGATTCCAAGG-3′	5′-CATGG**C**GCATTCTGCTTCTGC-3′
exon 2 – exon 3b	2	5′-ATGGCTCTCGTCTTCAGTGC-3′	5′-CTTCAGAGCCAAGACTATGA-3′
exon 9 – exon 24a	3	5′-CAACACTGTTACTGAAGTCGACAA-3′	5′-AAAGTGAAATGCCACAATCC-3
exon 8 – exon 11	4/5	5′-CTCAATACCAACCTGAGCTC-3′	5′-GGAGCTAATGGAGTCCAAGG-3′
exon 18 – exon 24a	6	5′-ACAGAATATAAGAGCCATCC-3′	5′-TAAAGTGAAATGCCACATCC-3′
exon 18 – exon 26	7/8	5′-ACAGAATATAAGAGCCATCC-3′	5′-CAACTCCAGTTGTCAGTATCGAG-3′
exon 19a – exon 23	8	5′-ATTTGTGAGGGTGAGGAATA-3′	5′-ACATCCTCTGAATCCATCCT-3′

### Protein isolation, immunoblotting and treatment of lysates with PNGaseF

For protein extraction, tissues were homogenized in lysis buffer (20 mM Tris HCl, pH 7.4, 137 mM NaCl, 10% Glycerin, 1% Triton-X-100, 2 mM EDTA, SPI and 1 mM PMSF). Protein amounts were measured by BCA protein assay according to manufacturer's protocol. For deglycosylation experiments, protein lysates (40 µg of protein) were incubated with glycoprotein denaturing buffer (5% SDS, 0.4 M DTT) (New England Biolabs #B1704S) for 10 min at 95°C. After denaturation, G7 buffer (#B3704S) and 10% NP-40 (#B2704S) were added to the samples. Subsequently, the samples were split in two. One aliquot treated with 500 units/µl PNGase F whereas the other served as an untreated control. Both aliquots were incubated overnight at 37°C. Per lane, 20 µg protein was loaded on 8% acryl amide gels and consecutively blotted onto PDVF membrane. Unspecific binding was blocked by 5% milk in PBS-0.1% Tween for 2 hours. Blots were incubated with either rat-anti-mouse prominin-1 (13A4, *e*Bioscience) or rabbit-anti-actin (I-19, sc-1616, Santa Cruz) overnight in blocking buffer, subsequently washed in PBS containing 0.2% Tween and incubated with HRP-labeled goat-anti-rat IgG (I1306, Santa Cruz Biotechnology) or goat anti-rabbit IgG (7074, Cell signaling) for 1 hour. For chemoluminescent visualization, ECL from Amersham Biosciences was used. The 13A4 blots were developed by the use of films, whereas the actin blots were developed by use of the FujiFilm LAS-3000.

### mRNA and putative promoter database and computer analyses

Analysis of mRNA and EST sequences was performed used the National Center for Biotechnology Information database (http://www.ncbi.nlm.nih.gov). Sequences were aligned in Vector NTI. Putative promoter regions were analyzed by comparing them to the genomice sequence (http://genome.ucsc.edu/). Potential transcription factor binding sites on the putative promoter regions were studied by using TFSearch (http://molsun1.cbrc.aist.go.jp/research/db/TFSEARCH.html) and Alibaba (http://www.gene-regulation.com/pub/programs/alibaba2/index.html). The putative promoter region was analyzed for a potential CpG island in CpG Island Searcher (http://cpgislands.usc.edu/cpg.aspx).

## Results

### Protein expression of prominin-1 splice variants in mouse tissues

**Figure 1:**
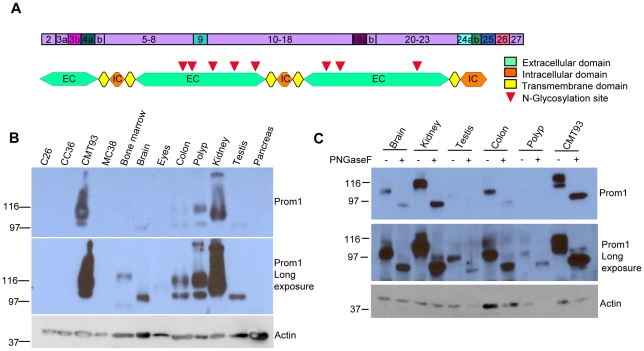
Expression of prominin 1 protein in several mouse tissues. **A.** Prominin-1 protein compared to the prominin-1 mRNA sequence. Indicated is where the spliced regions of the mRNA are localized on the protein. None of the N-linked glycosylation sites are localized in any the spliced exons. **B.** Immunoblot for prominin-1 on lysates of several mouse organs and murine CRC cell lines. As loading control, actin was used. **C.** Lysates of several mouse organs were deglycosylated with PNGaseF and compared to untreated controls.

Prominin-1 is a five transmembrane protein consisting of an extracellular N-terminal part, two large extracellular loops and an intracellular C-terminal tail. Extensive splicing of prominin-1 has been reported and this mostly affects the composition of intracellular C-terminal tail [17], [21]. In addition, some differentially spliced exons are localized in regions that are transcribed as part of the extracellular loops of the prominin-1 protein (Fig. 1A). Altogether, differential splicing results in distinct protein sizes, with different molecular weights and native iso-electric points (Table 2).

**Table 2:** mRNA and protein details of prominin-1 splice variants.

Splice variant	mRNA ORF (bp)	Amino acids	Protein (kD)	Isoelectric point
1	2576	858	96,24	6,50
2	2603	867	97,13	6,68
3	2504	834	93,46	6,48
4	2414	804	90,00	6,81
5	2429	809	90,62	6,70
6	2471	823	92,24	6,44
7	2483	827	92,73	6,54
8	2528	842	94,49	6,91

To gain more insight in the prominin-1 expression on protein, several organs from wild type C57Bl\6 mice, like brain, eyes, kidney, pancreas and testis and CRC cell lines like MC38, CC26, CC36 and CMT93 were lysed and protein samples were loaded on an immunoblot. Blotting with the 13A4 antibody detected the prominin-1 protein as one or multiple bands in these organs (Fig. 1B), ranging from ∼90 to 116 kD. The protein's apparent molecular weight was much higher than predicted as in Table 2. As prominin-1 is a highly glycosylated protein [17], [22] with eight potential N-linked glycosylation sites on two extracellular loops, the difference between the detected molecular weight and the predicted molecular weight was likely caused by glycosylation of the prominin-1 protein. Although differential splicing does not remove any N-linked glycosylation sites on the protein (Table 3), the glycosylation of the prominin-1 protein might be dependent on splicing and on the tissue in which it is expressed.

**Table 3:** Localization of N-glycosylation sites.

Glycosylation site	Amino acid location in prom1-SV2 (aa)	Loop location	Exon location
1	273	1	8
2	291	1	8
3	332	1	8
4	374	1	10
5	415	1	11
6	554	2	14
7	581	2	15
8	732	2	20

We therefore treated the lysates with PNGaseF to remove N-linked glycans and analyzed the prominin-1 detection on an immunoblot (Fig. 1C). Deglycosylation of the protein reduced, as expected, the molecular weight to 90–98 kD. In addition, multiple sizes of prominin-1 protein were visible on the blot, suggesting that different tissues express distinct SVs. In principle, it should be possible to discriminate between SV4/5 (∼90 kD), SV6/7 (∼92 kD), SV 3/8 (∼94 kD) and SV1/2 (∼97 kD) on a immunoblot, but in practice, this was difficult to achieve, since the difference in size were too small and different bands could be linked to several SVs.

Altogether, these data indicate that protein detection of prominin-1 SVs in different mouse tissue reveals the expression of different SVs. However, it is not a sufficiently discriminative approach to analyze differential usage of SVs. This is in part due to differential glycosylation, but also due to relatively similar sizes of some SVs after deglycosylation.

### Multiple prominin-1 splice variants are expressed in mouse organs

**Figure 2:**
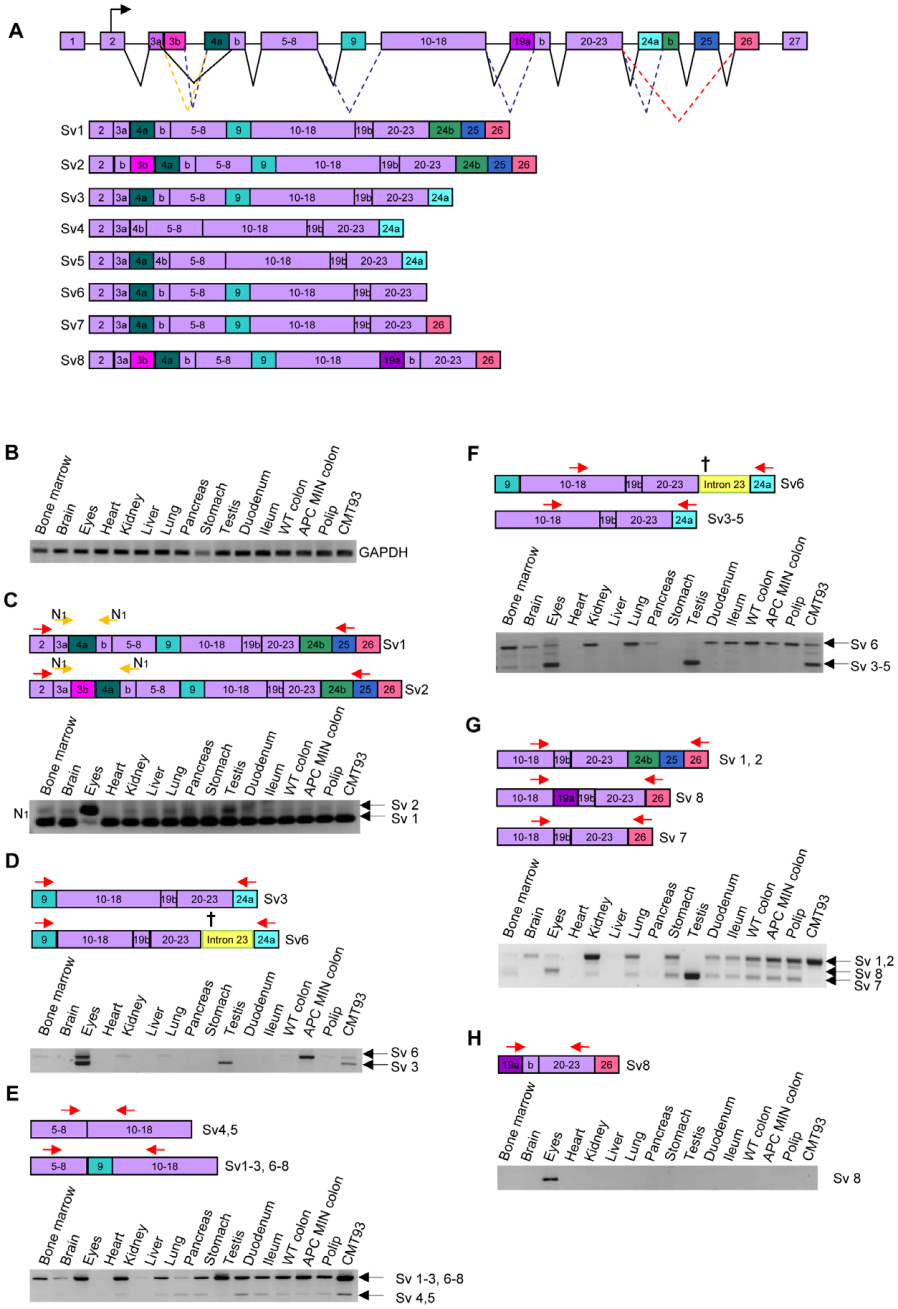
Expression of prominin-1 splice variants in mouse organs. **A.** Schematic representation of prominin-1 splicing. Differentially spliced exons are indicated by distinct colors. **B.** PCR performed on mouse tissues with primers amplifying GAPDH to confirm mRNA expression **C–H** PCR data representing prominin-1 SV expression in mouse tissues. Each subpanel contains the analysed SVs, the location were the primers bind (arrows) and the acquired products after performing the PCR. Primers sequences can be found in Table 3. **C.** PCR to specifically detect SV1 and SV2 with primers amplifying exon 2 to 25, only SV1 and SV2 products were obtained. Additionally, a nested PCR amplifying regions specific for SV1 and SV2 was performed. N1 and N2 stand for nested primer sets. **D.** SV3 was discriminated from the other SVs by amplifying the region from exon 9 to exon 24a. **E.** SV4 and SV5 were analysed together due to only a minor difference between these SVs (15 bp). By amplifying exon 8 to exon 11, SV4 and SV5 were discriminated from the other SVs. **F.** To specifically detect SV6, primers amplifying exon 18 to exon 24a were used, making use of the fact that SV6 retains intron 23. **G.** SV7 and SV8 were discriminated from the other SVs by amplifying exon 18 to exon 26. **H.** The expression pattern of SV8 was analyzed by using primers that bind the exon 19a, which is specifically expressed in SV8.

To obtain more insight into the prominin-1 SV expression in mouse tissues, we decided to study their expression on mRNA level. Aligning all mRNA and expressed sequence tags (ESTs) sequences of prominin-1 from the NCBI database, we confirmed the eight known SVs and their exon organization, as has been published before (Fig. 2A) [18]. To evaluate the expression of the different prominin-1 SVs in several tissues on mRNA level, we designed several distinct primer sets to specifically amplify the alternatively spliced regions (Table 3, Fig. 2C–H). We isolated mRNA from several mouse tissues like eye, heart, pancreas, testis and different parts of the gastrointestinal tract as confirmed by a GAPDH PCR (Fig. 2B).

SV1 and SV2 were discriminated from the other SVs by using primers that amplify the region from exon 2 to exon 25, which is specifically expressed in SV1/2 (Fig. 2C). To discriminate between SV1 and SV2, a nested PCR on this exon 2–25 PCR product was performed, amplifying exon 3a to 4b (Fig. 2C), because SV1 splices out exon 3b, whereas SV2 retains this exon. The data from this PCR showed that all tissues expressed SV1 as well as SV2, but the eyes showed the highest SV2 expression. Although this nested PCR is able to indicate if SV1 and SV2 are expressed in these tissues, the data can not be used to interpret differences in expression. This is illustrated by figure 2G, in which SV1 and SV2 are analyzed together in a direct PCR, which shows that there are quite some expression differences between tissues, e.g. there is very low expression of SV1/2 in the heart, liver and pancreas, whereas the kidney and all parts of the gastrointestinal tract highly express these SVs (Fig.2G).

The expression pattern of SV3 in mouse tissue was detected by amplifying exon 9 to exon 24a, which is specifically expressed in SV3 and the longer SV6 (Fig. 2D). Interestingly, the PCR data showed that SV3 is exclusively expressed by the eyes, the testis and CRC cell line CMT93 (Fig. 2D), suggesting that this SV has restricted expression in mouse tissues.

SV4 and SV5 were analyzed together, due to only a minor difference (15 bp) between the two variants. Also, these SVs have been shown earlier to be unable to reach the cell surface and therefore they are unlikely to have a prominent function. Primers amplifying the region from exon 8 to exon 11 only were designed to discriminate SV4/5 (which do not express exon 9) from the other SVs and the PCR data showed that SV4/5 were expressed to a low extend in all tissues (Fig. 2E). In addition, by comparing Figure 2F, in which we analyze SV3-5 together, with Figure 2D, in which only SV3 and SV6 were analyzed, we can conclude that the expression level of SV4/5 is very low, consistent with the idea that these would encode non-functional, potentially aberrant splice variants.

The SV6 expression pattern was analyzed by making use of the fact that this splice variant retains intron 23. The retention will induce an alternative stop codon, thereby inducing a shorter protein, although the mRNA sequence will be longer due to this retention. A PCR performed with primers amplifying exon 18 to exon 24a can therefore discriminate between SV6 and SV3-5 and showed that SV6 is expressed in a range of mouse tissues, but is virtually absent in the heart, liver, stomach and testis (Fig. 2F). These data are confirmed by the PCR performed in Figure 2D.

SV7 and 8 can be distinguished from the other SVs by using primers that amplify from exon 18 to exon 26, because only SV7 and SV8 express exon 26 without exon 24b and 25. To discriminate between SV7 and SV8, the PCR product includes the differentially spliced exon 19a. SV7 was found to be expressed by all tissues except for brain, heart, liver and pancreas (Fig. 2G). SV8 was only found to be expressed in the eye. A separate PCR was performed to confirm the SV8 expression profile, using primers to amplify exon 19 (specifically expressed by SV8) to exon 23. This also showed that SV8 was only expressed in the eyes (Fig. 2H).

Altogether, we can conclude from the prominin-1 mRNA SV expression pattern (Fig. 2, Table 4) that 1) the heart, liver and pancreas hardly express prominin-1; 2) SV1/2 are expressed in all other tissues, although at varying levels; 3) SV4/5 are hardly expressed in any of the tissues; 4) SV3 is only expressed in the eyes, testis and CRC cell line CMT93; 5) SV8 is uniquely expressed in the eyes; 6) the testis only expressed SV3 and SV7 and 7) the eye expressed SV2, SV3, SV6 and SV8. Altogether,this suggests that only SV3 and SV8 might have a more specific function in these tissues dependent on their splicing. The other SVs probably have either identical and/or redundant functions.

**Table 4:** Expression of prominin-1 splice variants in mouse tissues.

Organs	SV 1	SV 2	SV 3	SV 4&5	SV 6	SV 7	SV 8
Bone Marrow	**V**	**V**	**X**	**V**	**V**	**V**	**X**
Brain	**V**	**V**	**X**	**V**	**V**	**X**	**X**
Eyes	**V**	**V**	**V**	**V**	**V**	**X**	**V**
Heart	**V**	**V**	**X**	**V**	**X**	**X**	**X**
Kidney	**V**	**V**	**X**	**V**	**V**	**V**	**X**
Liver	**V**	**V**	**X**	**V**	**X**	**X**	**X**
Lung	**V**	**V**	**X**	**V**	**V**	**V**	**X**
Pancreas	**V**	**V**	**X**	**V**	**V**	**X**	**X**
Stomach	**V**	**V**	**X**	**V**	**X**	**V**	**X**
Testis	**V**	**V**	**V**	**V**	**X**	**V**	**X**
Duodenum	**V**	**V**	**X**	**V**	**V**	**V**	**X**
Ileum	**V**	**V**	**X**	**V**	**V**	**V**	**X**
WT colon	**V**	**V**	**X**	**V**	**V**	**V**	**X**
APC MIN colon	**V**	**V**	**X**	**V**	**V**	**V**	**X**
Polip	**V**	**V**	**X**	**V**	**V**	**V**	**X**
CMT93	**V**	**V**	**V**	**V**	**V**	**V**	**X**

Semi-quantitative analysis of prominin-1 splice variant PCR products. V means that the SV is expressed in the tissue, X means that no PCR product is observed.

### Putative promoter region of prominin-1

**Figure 3:**
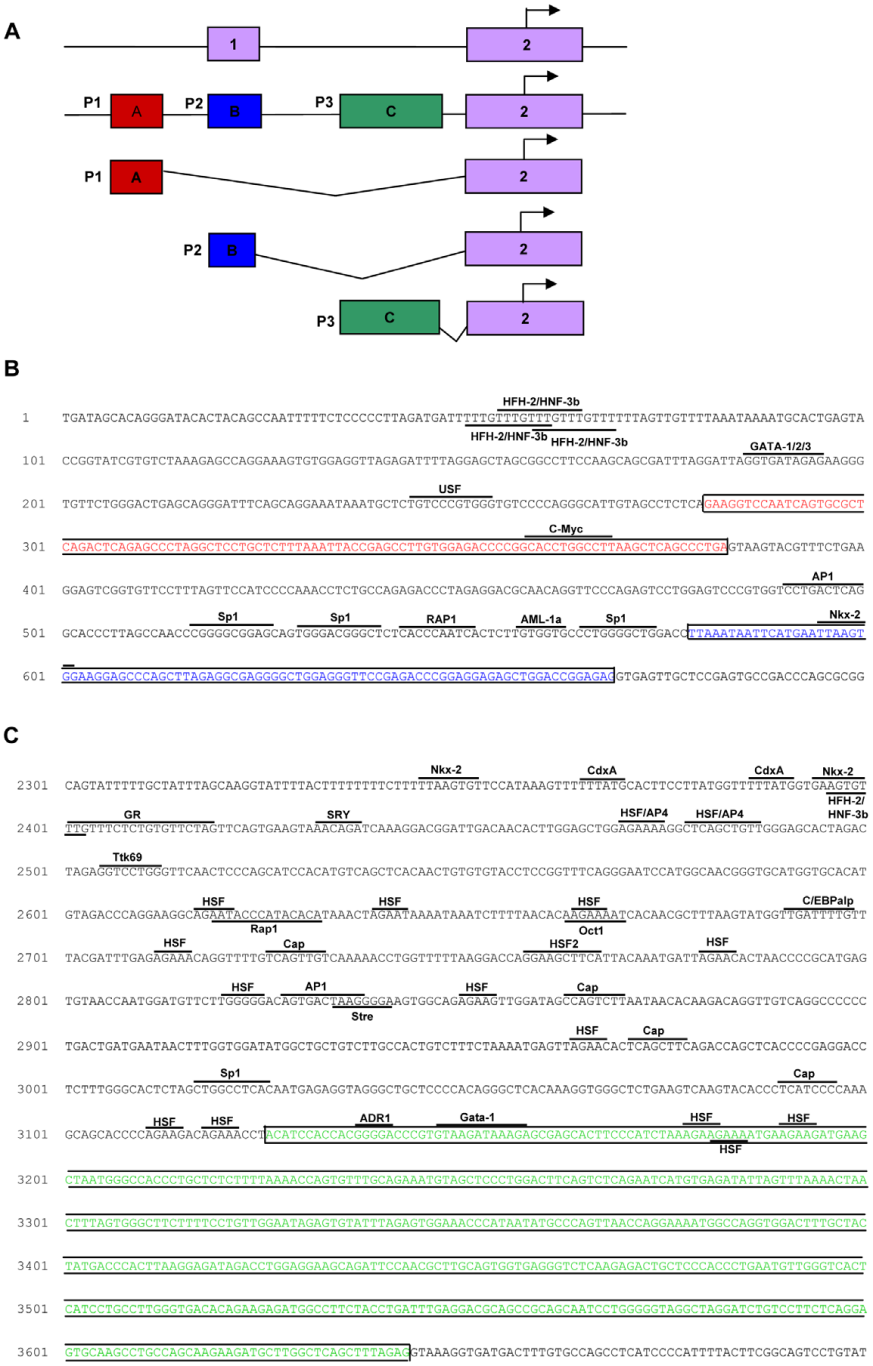
Putative promoter region of mouse prominin-1 with putative binding sites for transcription factors. **A.** Putative promoter regions found for the prominin-1 gene. The upper row indicates the exon organization as now found in the NCBI database, the other rows indicate the new putative promoter regions. **B.** Putative binding sites for transcription factors in putative promoter region 1 and 2, based upon database searching in TFSearch and Alibaba. **C.** Putative binding sites for transcription factors in putative promoter region 3, based upon database searching in TFSearch and Alibaba.

For human, it has been shown that multiple promoters exist that can drive the expression of prominin-1 [16], although no correlation was shown so far between specific promoter activity and distinct SV expression. We hypothesized that mouse prominin-1 is also regulated by several promoters. To this end, all 5′UTR of the different splice variants and the expressed sequence tags (ESTs) as described in the NCBI database were analyzed (Table 5). The alignment of the 5′UTRs to the genomic DNA revealed two extra exons in the 5′UTR region of the prominin-1 gene in addition to the previous identified exon (Fig. 3A). One exon (exon A in Fig. 3A) was found approximately 7.2 kb upstream of exon 2. This exon was specifically found in the 5′UTR of SV8. Exon 1, referred to as exon B in Fig. 3A, is located 6.9 kb upstream of exon 2, and seems to be mainly expressed in the 5′UTR of SV1 and SV2. A third exon (exon C), located 4.3 kb upstream of exon 2, was only found in the 5′UTR of SV7. SV3-6 did not contain a 5′UTR as described on the NCBI database and therefore, no additional 5′UTR exons and putative promoter regions could be identified for these SVs. Interestingly, two of the three found 5′UTRs were linked to SVs that show specific expression. Exon A was found in SV8, which is specifically expressed in the eye. Exon B was found in SV7, which is expressed in multiple tissues, but highly in the testis.

**Table 5:** Putative promoter region in prominin-1 mRNA and EST sequences.

Promoter region	mRNA sequences	EST sequences	SV	Expressed in
1	BC028286		SV8	Eye, retina
2	NM_001163577	BY036120	SV2	Brain- 8 week old
	NM_008935	BY063214	SV1	Kidney
	AF039663	BY094504		
	AF026269	BY105834		
		BY151317		
		BY304300		
3	AK029921		SV7	Testis
	AK030027			

Next, we analyzed if these putative promoter regions contain putative transcription factor binding sites, CpG islands or TATA box regions. No TATA boxes were found, but a potential CpG island was identified starting in putative promoter 2, spanning approximately 1500 bp (data not shown). In addition, many putative transcription factor binding sites were found in each of the putative promoter regions (Fig. 3B and C).

To conclude, specific 5′UTRs for SV1/2, SV7 and SV8 were found, suggesting that these SVs can be alternatively regulated by putative promoters. Different potential transcription factor binding sites were found in each of the potential promoter regions, indicating that the expression of these prominin-1 SVs might be differentially regulated.

## Discussion

In this study, we showed that several mouse organs express multiple prominin-1 splice variants on mRNA, indicating that these different variants probably have partially overlapping functions. The distinct expression of SV3 and SV8 compared to the other SVs hint towards a specific regulation of these SVs, which suggests that these SVS have a unique role compared to the other SVs. In literature, there are some indications that prominin-1 SV expression is regulated. For example, SV1 is downregulated in the brain during development, whereas SV3 is upregulated [21], hinting towards specific regulation of SV expression. In contrast, we did not detect SV3 expression in the adult brain. Although this SV is almost identical to SV6 on both mRNA and protein and could therefore easily been mistaken for SV6, our PCR strategy was designed as such that the 3′UTR of SV6, which is not present in SV3, could be indentified by PCR. Therefore we can conclude that SV6 is present in the brain and not SV3.

Although the exact function of prominin-1 and its SVs are thus far unknown, it has been shown to play an important role in morphogenesis of photoreceptor cells. For instance, patients that harbor mutations in the prominin-1 gene suffer from retinal degeneration [23], [24], [25]. In addition, prominin-1 deficient mice, which are viable and fertile, display blindness due to complete degeneration of mature photoreceptors [26]. Human CD133 was found to be important for morphogenesis of new disk membranes by interacting with actin (decreased by R373C mutation) and protocadherin 21 (PCDH21). One of the missense mutations found in patients with macular degeneration (R373C mutation) decreases this interaction between actin and CD133 and causes mislocalization of both CD133 and PCDH21 [25]. Interestingly, SV8 was only found to be expressed in the eye and in none of the other organs, which could potentially indicate that this SV may be of importance to the development of the eye. SV8 is the only SV that retains exon 19a, which is located in the second extracellular loop. In human, a similar CD133 SV has not been identified yet.

As been shown for human [16], we found several distinct putative promoter regions for the mouse prominin-1 gene. Although the functionality of these promoters remains to be defined, the specific region found in the 5′UTR of SV8, the SV that is only expressed in the eye, hints towards a selective regulation of this prominin-1 SV expression by an eye-selective promoter. In addition, as shown for human CD133 promoters [27], [28] , regulation by methylation could explain for example the selective expression of SV8 in the eye. Also, the many putative binding sites for the transcription factor ‘heat shock factor’ (HSF) in the putative promoter region 3, which appears to be linked to the on testis highly expressed SV7, are striking. Especially as HSF is known to be involved in chromatin reorganization in sperm [29], [30], suggesting that this transcription factor might regulate prominin-1 SV7 expression in the testis.

We have shown that most of prominin-1 SVs are differentially expressed in the majority of mouse tissues. Especially, SV3 and SV8 expression is retained to specific tissues. Further studies will have to reveal if these SVs have the capacity to interact with specific binding partners or induce specific signaling. Specific prominin-1 SV expression combined with selective promoter usage hints towards a complex regulation of prominin-1 in mice, as was found for humans. Additional studies, revealing the function, ligand and/or binding partners of prominin-1 will shed more light on the possible function of this heavy regulation.
